# Simultaneous thrombosis of multiple coronary arteries in a patient with rheumatoid arthritis

**DOI:** 10.15171/jcvtr.2016.17

**Published:** 2016-06-28

**Authors:** Arzu Kalayci, Erol Arslan, Salih Murat Bakar, Mahmut Guneri, Rafet Dizman, Eylem Kivanc, Can Yucel Karabay

**Affiliations:** ^1^Yunus Emre State Hospital, Eskisehir, Turkey

**Keywords:** Rheumatoid arthritis, Methotrexate, Coronary thrombosis

## Abstract

We present a case of simultaneous coronary thrombosis of the left main, the left anterior descending artery and the right coronary artery in a patient, recently diagnosed with rheumatoid arthritis.

## Case Report

**Figure 1:**
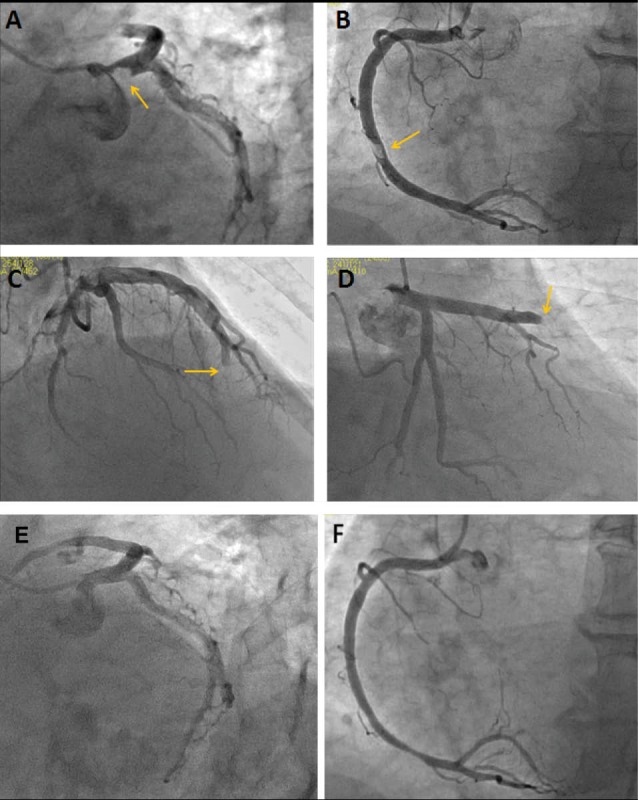
Diagnostic coronary angiogram shows an ostial LMCA thrombus with 90% obstruction (A), a total occlusion of the mid left anterior descending artery (C,D) and a mid right coronary artery (RCA) thrombus with 80% obstruction (B). Control coronary angiogram demonstrated complete dissolution of the large thrombus in ostial LMCA (E) and the mid portion of RCA (F) and the residual occlusion of the apical portion of the LAD (E).

A 62-year-old male without known coronary artery disease, recently diagnosed with rheumatoid arthritis (RA) treated with methotrexate for 5 days, was admitted to our emergency department with acute and typical chest pain for an hour. On ECG, ST-segment elevation myocardial infarction (STEMI) in anterior and inferior leads were noted. There were hypertension and hyperlipidemia as risk factors of coronary artery disease. After the patient was treated with 300 mg of aspirin, 600 mg of clopidogrel, as well as a weight-adjusted (70 IU/kg) bolus of unfractionated heparin, coronary angiography was performed showing an ostial left main coronary artery (LMCA) thrombus with 90% obstruction (Figure 1A, supplementary file 1), a total occlusion of the mid left anterior descending artery (LAD) (Figure 1C, 1D) and a mid right coronary artery (RCA) thrombus with 80% obstruction (Figure 1B, supplementary file 2). From this point on we had three possible strategies. The first one was the interventional strategy (stenting/aspiration with high risk of cerebral and peripheral embolism, which would have exposed the patient to short and long-term risks). The second one was the coronary artery bypass graft surgery (the coronary artery bypass grafting may represent a high risk of early occlusion because of normal native coronary flow after dissolving the coronary thrombus with medical therapy) and the third one was pharmacological strategy (thrombolytic therapy), eventually followed by the pharmacological strategy. Thus, based on the STEMI guideline of European Society of Cardiology; alteplase (rt-PA) 15 mg was injected intravenosus as a bolus dose and subsequently 50 mg alteplase was infused for 30 minutes (0.75 mg/min) and 35 mg was infused for 60 minutes. After the fibrinolytic therapy, clinical and electrocardiographic success criteria were obtained. Repeated coronary angiography was performed 24 hours later, revealing that the large thrombus in ostial LMCA (Figure 1E) and mid portion of RCA (Figure 1F) was totally dissolved but only the apical portion of the LAD was occluded (Figure 1E). In addition, laboratory workup for hypercoagulability was negative.

## Discussion

It is well known that patients with RA, as well as other autoimmune diseases, have an increased incidence of cardiovascular disease compared to the general population. This increased incidence is mainly due to endothelial dysfunction, accelerated coronary artery disease and increased incidence of metabolic syndrome.^1^ It has also been suggested by several studies that RA confers a prothrombotic state based on abnormalities in coagulation and fibrinolytic systems together with a variable state of platelet functions in several studies. High level of C-reactive protein, thrombocytosis, hyperfibrinogenemia, high activity of Von Willebrand factor, a low level of antithrombin III, suppression of fibrinolysis in blood plasma, antiphospholipid antibody positivity, and high levels of plasma homocysteine have all been reported in RA patients. Furthermore, chronic inflammation induced by RA can cause endothelial cell activation and vascular dysfunction.^2^

In the present case, although RA per se may have been the underlying etiology for coronary thrombosis, we need to take in to account recently administrated Methotrexate therapy. In current studies, vascular toxicity and thrombotic effects has been reported with some antineoplastic agents. Thus, it is important for clinicians to be aware of infrequent and different, but potentially serious, adverse cardiac effects of these agents.

Methotrexate, a synthetic folic acid analogue, is an antineoplastic and immunomodulating compound that has gained wide acceptance in the management of rheumatoid artritis, psoriasis, sarcoidosis and a number of neoplastic disorders.^3^ The most common serious adverse reaction seen with methotrexate is hepatotoxicity; however, a number of other serious types of adverse events has been reported including hematologic, gastrointestinal, cardiovascular, renal, dermatological, pulmonary, neurologic, and immunologic events.^3-6^ In a retrospective review of methotrexate use in clinical practice, 50% of patients reported an adverse event while on therapy, with 32% of those being classified as ‘significant’.^7^ There are a few case reports in the literature showed cerebral venous thrombosis after intrathecal methotrexate administration^8^ and there is a case report showed acute coronary thrombosis and myocardial ischemia following combined chemotherapy including methotrexate in Hodgkin›s disease.^9^

On the other hand, potential cardioprotective effect of methotrexate was investigated currently in some trials: the TETHYS trial^10^ revealed the anti-inflammatory and anti-ischemic effects of methotrexate in patients with acute myocardial infarction. However, the CIRT trial^11^ demonstrated low-dose methotrexate reduces heart attacks, strokes, or death in people with type 2 diabetes or metabolic syndrome. Effective suppression of systemic inflammation can be seen as a method for prevention cardiovascular diseases.

Although, methotrexate therapy has some cardioprotective effects, it might lead to increased tendency towards thrombus formation.

In conclusion, we present a case of simultaneous coronary thrombosis of the left main, the LAD artery and the RCA in a patient, recently diagnosed with RA.

## Ethical approval

This study was approved by the local committee of ethics.

## Competing interests

Authors declare no conflict of interests in this study.

## Supplementary materials

Supplementary files 1

Supplementary files 2 consist of videos 1 and 2, respectively.
